# Understanding the Radiation Dose Variability in Nasopharyngeal Cancer: An Organs-at-Risk Approach

**DOI:** 10.7759/cureus.49882

**Published:** 2023-12-03

**Authors:** Abdulrahman Bin Sumaida, Nandan M Shanbhag, Khalid S Balaraj, Rajmane Puratchipithan, Syed Mansoor Hasnain, Omran El-Koha, Amjad Hussain, Theresa Binz, Vivek T Rajendran, Rajaneesh Kumar R Nair, Noor H Jaafar, Mohammad Saleh, Khaled Al Qawasmeh

**Affiliations:** 1 Oncology/Radiation Oncology, Tawam Hospital, Al Ain, ARE; 2 Oncology/Palliative Care, Tawam Hospital, Al Ain, ARE; 3 Internal Medicine, United Arab Emirates University, Al Ain, ARE; 4 Oncology, Tawam Hospital, Al Ain, ARE; 5 Radiation Oncology, Tawam Hospital, Al Ain, ARE; 6 Medical Physics, Tawam Hospital, Al Ain, ARE; 7 Radiotherapy Technology, Tawam Hospital, Al Ain, ARE; 8 Nursing, Tawam Hospital, Al Ain, ARE

**Keywords:** nasopharyngeal cancer, brainstem, organs at risk, predictive modeling, radiation oncology

## Abstract

Objective

This study aims to thoroughly assess the radiation dose distribution to critical organs in patients with nasopharyngeal carcinoma, focusing on the correlation between the radiation dosages for the various organs at risk (OARs) in nasopharyngeal cancer patients.

Methods

We meticulously analysed a dataset comprising 38 nasopharyngeal carcinoma patients, focusing on radiation dosages measured in Gray (Gy) and volumetric data in cubic centimetres (cc) of critical organs, including the lens, brainstem, spinal cord, optic nerve, optic chiasm, and cochlea. A detailed exploratory data analysis approach encompassed univariate, bivariate, and multivariate techniques.

Results

Our analysis revealed several key findings. The mean and median values across various dose measurements were closely aligned, indicating symmetrical distributions with minimal skewness. The histograms further corroborated this, showing evenly distributed dose values across different anatomical regions. The correlation matrix highlighted varying degrees of interrelationships between the doses, with some showing strong correlations while others exhibited minimal or no correlation. The 3D scatter plot provided a view of the multi-dimensional dose relationships, with a specific focus on the spinal cord, lens, and brainstem doses. The bivariate scatter plots revealed symmetrical distributions between the right and left lens doses and more complex relationships involving the brainstem and spinal cord, illustrating the intricacies of dose distribution in radiation therapy.

Conclusion

Our findings reveal distinct radiation exposure patterns to OARs of nasopharyngeal carcinoma. This research emphasises the need for tailored radiation therapy planning to achieve optimal clinical outcomes while safeguarding vital organs.

## Introduction

Nasopharyngeal cancer (NPC) is the most unique of all head and neck cancers and is characterised by its distinct geographic and demographic distribution. Globally, the incidence of NPC varies significantly, with rates ranging between 6.4 (Southeast Asia) and 0.2 (Europe) per 100,000 and a threefold higher incidence in men compared to women [1]. In 2019 more than 176,501 new cases were reported worldwide, indicating an age-standardized incidence rate of 2.1 [2].

In the United Arab Emirates (UAE), the incidence of NPC is notably lower, generally well below 1.0 per 100,000 per annum [3]. This lower incidence in the UAE aligns with global patterns, where Western and Middle Eastern countries typically report lower NPC rates than East and Southeast Asian countries.

Radiotherapy is critical in treating NPC, offering a potential cure, especially early-stage disease. The proximity of nasopharyngeal tumours to essential structures of the head and neck area, such as the brain, spinal cord, and salivary glands, makes treatment planning complex. Identifying and preserving the integrity of these organs at risk (OARs) during radiotherapy is crucial to minimise treatment-related morbidity while maximising therapeutic efficacy [4].

The landscape of NPC radiotherapy has evolved significantly over the years, incorporating a range of technological advancements to improve patient outcomes. These innovations include advanced imaging modalities for precise tumour targeting and the standardisation of treatment planning. This has been augmented by adaptive re-planning techniques, particle therapy, and artificial intelligence integration to optimise treatment strategies [5].

Recent developments have also focused on improving the radiation dose and target delineation to maximise the benefits of radiotherapy while minimising its side effects. This has been particularly important for preserving the function of OARs in the head and neck region [6]. Despite these advances, managing distant failure remains a key challenge in treating NPC, highlighting the need for ongoing research and development [7].

Concurrent chemoradiotherapy, conformal and intensity-modulated radiotherapy (IMRT), and salvage options for locoregional recurrence represent significant steps forward in NPC management. These approaches have improved the prognosis for patients with NPC, achieving better tumour control and enhancing overall survival rates [8]. The integration of chemotherapy (neo-adjuvant and concurrent) with radiotherapy has been particularly impactful, demonstrating high response rates in local and regionally advanced disease scenarios [9]. In this context, this study aims to enhance the precision and efficacy of radiation therapy by providing detailed insights into the distribution and interrelation of radiation doses across various critical anatomical regions.

## Materials and methods

This retrospective study included 38 of 41 patients diagnosed with nasopharyngeal carcinoma. These patients varied in age from 10 to 74 years, with an average age of 40.51 years, and comprised 31 males and seven females. The predominant radiation treatment modality was tomotherapy, used in 89% of cases with the simultaneous integrated boost (SIB) technique. The treatment volume included the primary treated to a dose of 70Gy in 2Gy per fraction and the neck treated as low-risk, high-risk, and involved areas. Thirty-six patients in the cohort were node-positive (at least stage III per the tumour, node, and metastasis staging), and two patients were node-negative. Most patients underwent induction chemotherapy before receiving concurrent chemo-radiation. The dataset included comprehensive treatment and dosimetric data, capturing radiation doses measured in Gray (Gy) and volumes in cubic centimetres (cc) for various OAR, including the lens, brainstem, spinal cord, optic nerve, optic chiasm, and cochlea (Table 1).

**Table 1:** Organs at Risk with Dose in Gy and Volume in cc n = 38, Gy: Gray, cc: cubic centimetres, std: standard deviation, min: minimum, max: maximum

Organs at Risk	Min.	Max.	Mean	Std.	Median
Lens Left Max Dose in Gy	5.09	12.211	8.081	1.8508	7.837
Lens Left Mean Dose in Gy	3.46	8.26	5.734	1.2829	5.668
Lens Right Max Dose in Gy	5.13	12.75	8.323	1.8343	8.329
Lens Right Mean Dose in Gy	3.47	7.68	5.737	1.2007	5.772
Brainstem Max Dose in Gy	40.4	62.34	56.012	4.2523	57.017
Brainstem Mean Dose Gy	19.24	37.03	27.021	4.1837	25.605
Spinal Cord Max Dose in Gy	19.42	46.56	30.793	5.8867	30.595
Spinal Cord Mean Dose in Gy	7.89	26.35	13.927	4.2052	13.805
Optic Nerve Right Max Dose in Gy	23.74	55.47	48.024	6.7435	48.985
Optic Nerve Right Mean Dose in Gy	13.6	45.61	30.915	7.0044	32.055
Optic Nerve Left Max Dose in Gy	21.3	57.49	48.661	7.2498	50.395
Optic Nerve Left Mean Dose in Gy	15.13	42.9	31.465	6.7735	32.181
Optic Chiasm Max Dose in Gy	21.19	55.26	45.969	6.874	47.598
Optic Chiasm Mean Dose in Gy	14.71	52.25	35.316	7.8337	36.321
Cochlea Left Max Dose in Gy	34.92	66.39	56.559	7.6301	58.691
Cochlea Left Mean Dose in Gy	22.47	56.98	42.691	8.4429	44.998
Cochlea Right Max Dose in Gy	34.92	69.9	58.252	8.0912	60.239
Cochlea Right Mean Dose in Gy	22.47	60.43	43.943	8.9266	44.803
Cochlea Left Volume cc	0.1	0.88	0.447	0.1784	
Cochlea Right Volume cc	0.1	1.2	0.465	0.2012	

Data were sourced from patient records at the Department of Radiation Oncology, Tawam Hospital, with the study period spanning from 2016 to 2022.

Data analysis

Before analysis, the dataset underwent meticulous preprocessing to ensure accuracy and relevance. This included the verification of data integrity, normalization of dosimetric values, and handling of missing data. Special attention was given to handling non-numeric entries and outliers to maintain the integrity of the statistical analysis. Standardization procedures were employed to bring all variables to a comparable scale, facilitating more accurate and meaningful comparisons across different dosimetric parameters.

Data preprocessing and analysis were conducted using Python with essential libraries, including Pandas and NumPy, for data manipulation and numerical computations. SciPy was used for statistical analysis, including the Mann-Whitney U test. Matplotlib and Seaborn were used for data visualisation, including histograms, boxplots, scatter plots, and heat maps.

The dataset initially contained 41 patients with a small percentage of missing values across several variables. To maintain the integrity and accuracy of the analysis, a decision was made to handle these missing data points through removal rather than imputation. This approach was deemed appropriate given the relatively low proportion of missing values (ranging from approximately 2.63% to 5.26% in affected columns) and the critical nature of the data in the context of radiation therapy planning.

Each row containing missing values was carefully reviewed and removed from the dataset. This method of handling missing data ensured that the statistical analyses were conducted on a dataset comprising only complete cases. While this approach might slightly reduce the sample size, it was chosen to avoid the potential biases and inaccuracies that could arise from imputation in a dataset where precise dosimetric data is paramount.

The removal of cases with missing data was conducted before any statistical analyses, including univariate, bivariate, and multivariate analyses. This ensured that all analyses were based on the most accurate and complete data available, thereby enhancing the validity and reliability of the study's findings.

Statistical analysis

Descriptive statistics were computed to summarise the dataset's distribution's central tendency, dispersion, and shape. Bivariate analysis, including Pearson and Spearman correlation tests, explored relationships between variables; non-parametric tests (Mann-Whitney U) were employed for variables not following a normal distribution.

Ethical considerations

The Institutional Review Board (IRB) of Tawam Hospital - Tawam Human Research Ethics Committee reviewed and approved the study protocol with approval number MF2058-2023-969. Patient confidentiality and data privacy were strictly maintained, with all identifiers removed before analysis.

## Results

**Figure 1:**
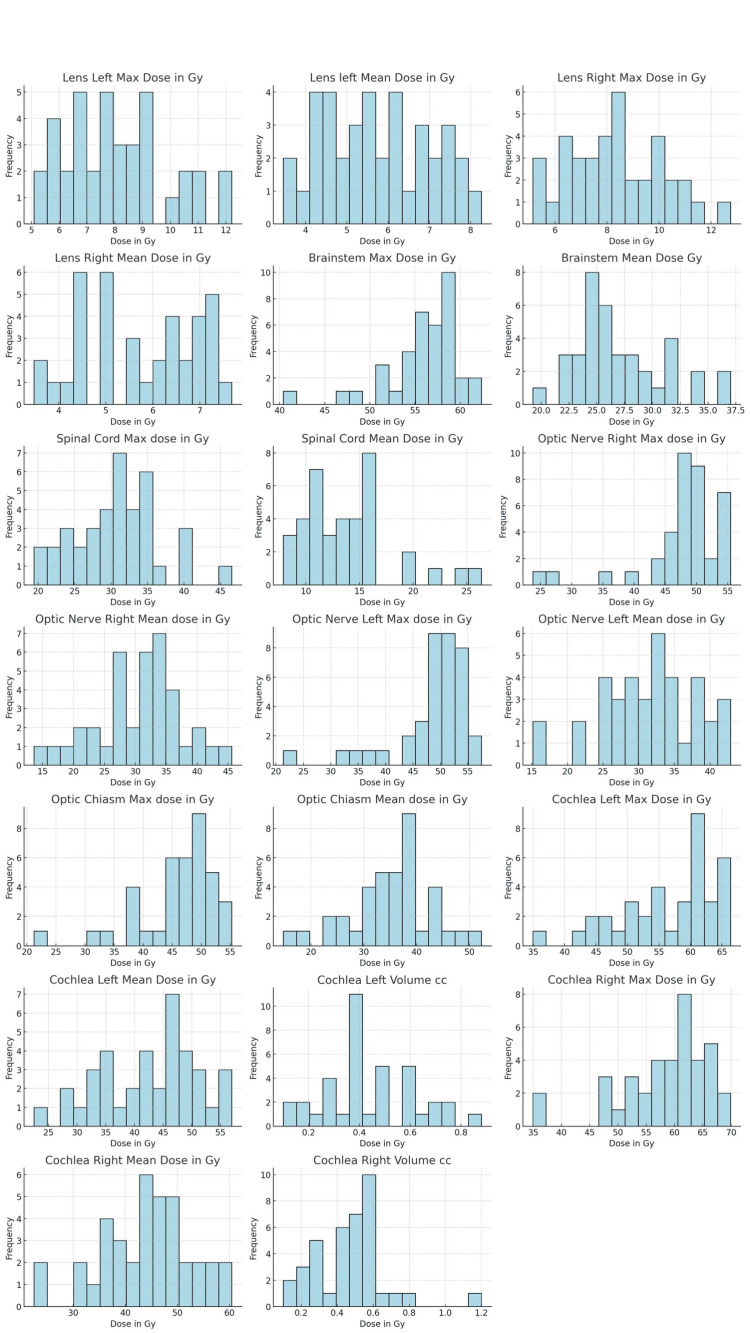
Distribution of Radiation Dosages and Volumes in Nasopharyngeal Cancer Patients Gy: Gray, cc: cubic centimetres

The analysis of radiation dosages and volumes revealed through histograms highlights distinct patterns in frequency distribution across different variables (Figure 1). The dataset's mean and median appear to be close to each other, indicating that the dataset was, for the most part, following normal distribution (Table 1).

Each histogram corresponds to a specific variable, such as radiation dose in Gy or organ volume in cc. The X-axis of these histograms represents the range of values for each variable, while the Y-axis shows the frequency of these values within the patient dataset. Notably, the distribution of values varies significantly across different organs. For example, the "Brainstem Max Dose in Gy" demonstrates a broad spread of values, indicating a diverse range of radiation doses patients received. In contrast, variables like the "Lens Left Max Dose in Gy" show a more concentrated distribution, suggesting consistency in the dosages administered to this OAR.

Bivariate analysis

**Figure 2:**
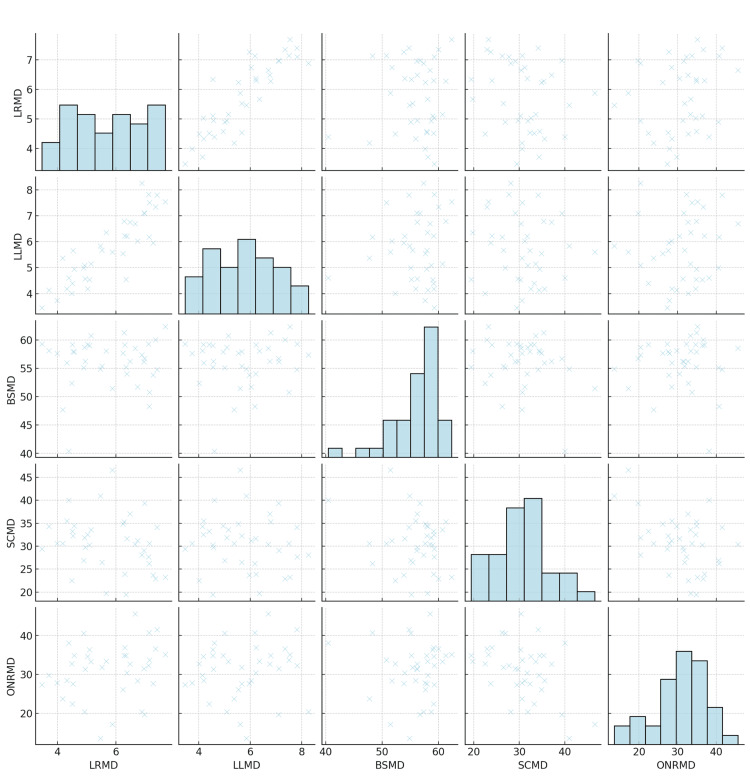
Scatter Plots to Compare Pairs of Variables LRMD: Lens Right Mean Dose in Gy, LLMD: Lens Left Mean Dose in Gy, BSMD: Brainstem Max Dose in Gy, SCMD: Spinal Cord Max Dose in Gy, ONRMD: Optic Nerve Right Mean Dose in Gy, Gy: Gray

The bivariate analysis using scatter plots explains the relationships between selected dosimetric parameters. The scatter plots compare pairs of variables, revealing patterns and correlations (Figure 2).

Lens Right Mean Dose and Lens Left Mean Dose (LRMD vs. LLMD)

This plot exhibits a strong positive linear correlation, as indicated by the dense clustering of points along a diagonal line. The similarity in dosages between the right and left lenses suggests a symmetrical approach in radiation therapy for these organs. The symmetrical dosages between the right and left lenses in radiation therapy indicate a precise and balanced treatment approach, crucial for minimizing ocular complications and ensuring uniform protection of these sensitive organs.

Brainstem Max Dose and Lens Left Mean Dose (BSMD vs. LLMD)

The points are more dispersed, indicating a weaker correlation. This dispersion suggests that the dosage to the brainstem does not consistently correlate with the dosage to the left lens, reflecting individualised treatment planning.

Brainstem Max Dose and Lens Right Mean Dose (BSMD vs. LRMD)

Similar to the previous plot, this graph shows dispersed points, underscoring the individualised approach in balancing brainstem protection with effective treatment.

Spinal Cord Max Dose and Lens Left Mean Dose (SCMD vs. LLMD)

The scatter plot reveals a weak negative correlation, as evident from the slight downward trend of the points. This suggests that higher doses to the spinal cord are not typically associated with higher doses to the left lens.

Optic Nerve Right Mean Dose and Lens Right Mean Dose (ONRMD vs. LRMD)

The plot indicates a moderate positive correlation. This relationship implies that dosages to the optic nerve and the right lens are related, possibly due to their anatomical proximity.

The scatter plots illustrate the complex interplay between radiation doses to various OARs. While there is an apparent symmetry in treatment for bilateral organs (lens), the relationships involving critical structures like the brainstem and spinal cord are less straightforward, highlighting the tailored nature of radiation therapy.

**Figure 3:**
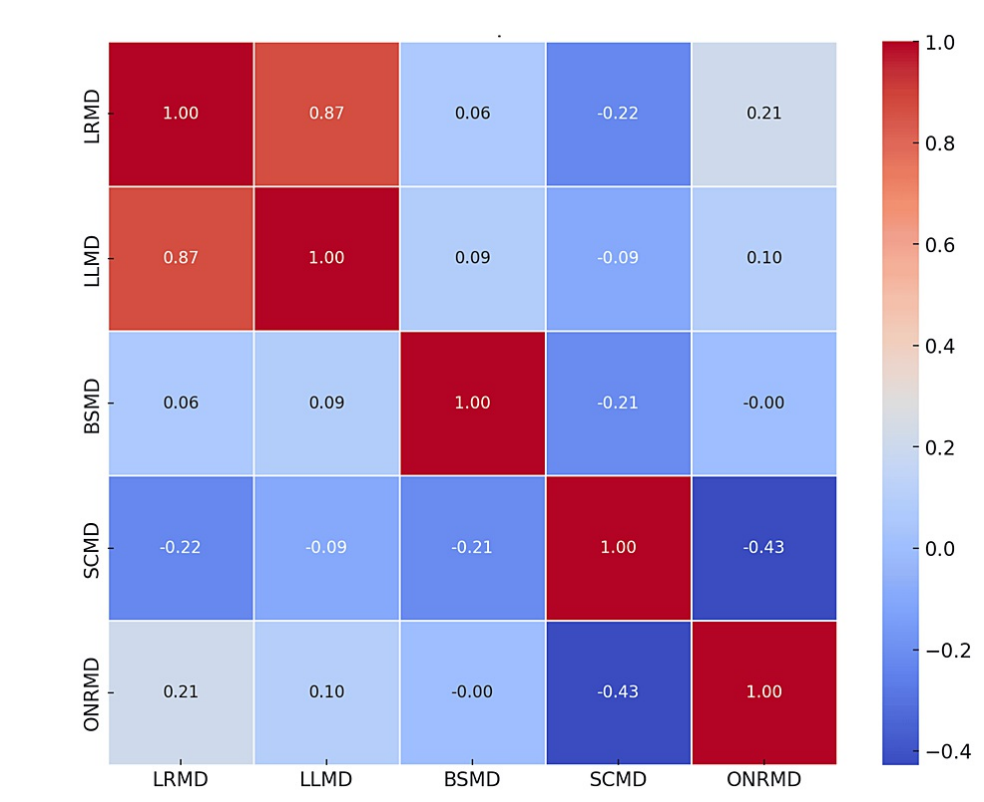
Correlation Matrix LRMD: Lens Right Mean Dose in Gy, LLMD: Lens Left Mean Dose in Gy, BSMD: Brainstem Max Dose in Gy, SCMD: Spinal Cord Max Dose in Gy, ONRMD: Optic Nerve Right Mean Dose in Gy, Gy: Gray

The correlation matrix quantifies the relationships (Figure 3).

High positive values indicate strong correlations, while values close to zero suggest weak or no correlation. Negative values indicate inverse relationships.

The strongest positive correlation is between LRMD and LLMD (0.87), indicating similar dosages for both the right and left lenses. A weaker negative correlation is observed between BSMD (in Gy) and SCMD (in Gy) at -0.21, suggesting variability in the dosages for these critical structures.

**Figure 4:**
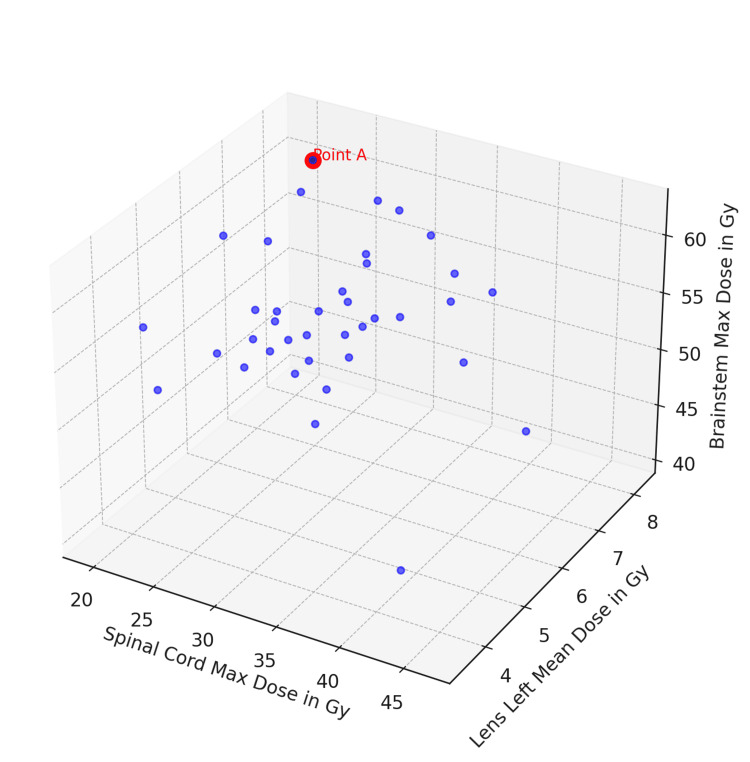
3D Plot of Key Dosimetric Parameters in Nasopharyngeal Cancer Patients X-axis: Spinal Cord Max Dose in Gy, Y-axis: Lens Left Mean Dose in Gy, Z-axis: Brainstem Max Dose in Gy

To offer a multi-dimensional perspective of the interplay between three structures, a 3-dimensional plot was charted (Figure 4).

Each point in the plot represents a patient's treatment data, showcasing the variability and relationship between spinal cord dose, lens dose, and brainstem dose. As an example, Point A represents SCMD in Gy (X-axis): 23.215 Gy, LLMD in Gy (Y-axis, renamed): 7.539 Gy and BMD in Gy (Z-axis): 62.345 Gy. There is considerable variability in the doses received by the spinal cord, lens, and brainstem across patients. This variability could be due to individual differences in anatomy, tumour location and size, and specific clinical objectives for each patient. The absence of distinct clusters suggests that there isn’t a one-size-fits-all approach to dosing. Instead, each patient’s treatment plan appears to be highly individualised, balancing the need to control the tumour with minimising damage to critical structures.

**Figure 5:**
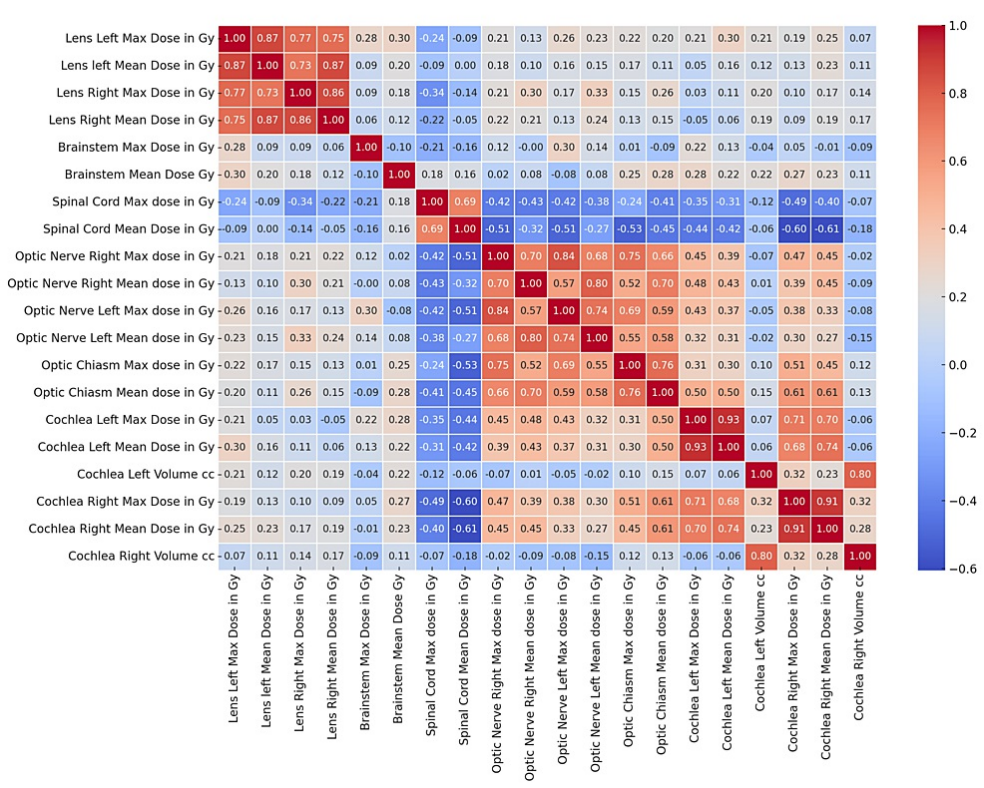
Correlation Heatmap of Nasopharynx Organs at Risk Gy: Gray, cc: cubic centimetres

To understand the relationships between all the variables in the study, a correlation heatmap (Figure 5) captures this well.

Specific pairs of variables show high positive correlations, indicated by darker red colours. These pairs have correlation coefficients close to 1.0, suggesting a strong direct relationship where the other increases as one variable increases. Some pairs exhibit negative correlations (darker blue areas), indicating an inverse relationship where an increase in one variable is associated with a decrease in the other. Many pairs of variables have moderate to low correlations, as indicated by lighter shades of red and blue. These relationships are less pronounced, suggesting a weaker link between the variables. Areas with very light colours (close to white) denote little correlation, implying that changes in one variable do not reliably predict changes in the other.

## Discussion

Analysing radiation dosages and volumes across different OARs in NPC patients highlights significant individual variability. This variation is likely due to multiple factors, including differences in tumour size, location, patient anatomy, and specific clinical objectives. For instance, the brainstem and lenses receive different radiation doses, reflecting the complex nature of treatment planning and execution. Studies have shown that while MRI images can delineate the boundaries of critical structures like the brainstem and optic nerve, there is no consistent correlation between the radiation doses to the lens and other OAR in NPC patients [10].

The correlation analysis between different OARs reveals insights into radiation therapy's complexity. For example, the positive correlation between right and left lens doses suggests a balanced approach to protect these symmetrical organs. However, the weaker correlations involving the brainstem and spinal cord doses highlight the individualised nature of treatment plans. Each patient's treatment is adapted to their unique situation, aiming to minimise the risk of injury to these critical structures. This individualisation is crucial, as research has shown a significant association between the dose to the brainstem and radiation-induced damage, emphasising the need for careful dose management [11].

The study's findings on radiation doses have important implications for patient outcomes. High radiation doses to sensitive structures can lead to significant side effects. For instance, the association between radiation dose to the brainstem and cerebellum and patient fatigue illustrates the clinical impact of radiation therapy beyond tumour control [12]. Radiation-induced necrosis has been reported in studies where the dose to the critical nerve tissue is beyond the threshold [13].

Radiotherapy may also cause hypothyroidism, with different rates of development in various patient subgroups. Monitoring thyroid function is essential in the follow-up period [14].

Radiotherapy can cause severe damage to the optic nerve, potentially leading to rapid visual deterioration or even blindness. Radiation-induced optic neuropathy (RION) is a rare but devastating complication causing rapidly progressive blindness in one or both eyes [15]. In addition, it can cause significant injury to the lens, leading to conditions like cataracts, and may also affect the retina and lacrimal apparatus. The risks of severe dry-eye syndrome, retinopathy, and optic neuropathy increase with max doses above 40, 50, and 60 Gy, respectively [16].

Increased doses to the cochlea and retro-cochlear auditory pathways are at risk for developing persistent sensorineural hearing loss. This effect is seen in a notable proportion of patients even after a median follow-up of two years [17,18].

Thus, while aiming for effective tumour eradication, the radiation therapy plan must also prioritise minimising adverse effects to enhance patient quality of life.

The study highlights the critical importance of personalised treatment planning in NPC radiotherapy. Given the proximity of nasopharyngeal tumours to critical head and neck structures, there is a fine line between delivering effective doses to the tumour and safeguarding the surrounding healthy tissue. Personalised treatment plans, informed by detailed imaging and patient-specific data, are vital to achieving this balance. This approach not only maximises the chances of successful treatment but also plays a crucial role in reducing the risk of long-term complications and improving the patient's quality of life.

Limitations of the study

The study was based on a relatively small sample of 38 patients. Such a sample size may limit the research's statistical power and the findings' generalizability. More extensive studies are needed to confirm these results and ensure they apply to a broader NPC patient population.

The study is subject to inherent selection and information biases as a retrospective analysis.

The absence of a control group in the study design limits the ability to make definitive conclusions about the efficacy and safety of the treatment approaches. A control group, ideally in a randomised controlled trial, would provide a benchmark for comparing outcomes.

The study included patients with varying stages of NPC, which could introduce heterogeneity in tumour characteristics, treatment responses, and outcomes. This variability might affect the reliability of the correlations and patterns observed in the study.

Being conducted in a single hospital, the study's findings may be influenced by the specific patient population, clinical practices, and technology available at that centre. Multi-centre studies would help validate the findings across different settings and populations.

The study focused on radiation dosimetry and did not extensively explore other treatment factors like chemotherapy regimens, patient comorbidities, or lifestyle factors that could significantly impact treatment outcomes.

The study's findings are based on the technology and treatment protocols available during the study period (2016-2022 with most patients treated with tomotherapy). Rapid advancements in medical technology and treatment strategies may limit the relevance of these findings in the future.

## Conclusions

This study, encompassing a detailed analysis of 38 NPC patients, offers significant insights into the radiation dosages delivered to various OARs. Our exploration contained univariate, bivariate, and multivariate analyses, providing a comprehensive view of the treatment landscape in this patient cohort.

In conclusion, this study emphasises the importance of personalised treatment planning in radiation therapy for NPC. The insights gained from the analysis enhance our understanding of radiation dosage patterns and highlight the need for meticulous planning to balance treatment efficacy with minimising risk to critical organs. The findings serve as a foundation for further research and development in the field, aiming towards optimised, patient-centric cancer care.

## References

[1] Gatta G (2021). Epidemiological aspects in nasopharyngeal cancer. Critical Issues in Head and Neck Oncology.

[2] Song Y, Cheng W, Li H, Liu X (2022). The global, regional, national burden of nasopharyngeal cancer and its attributable risk factors (1990-2019) and predictions to 2035. Cancer Med.

[3] (2023). The Global Cancer Observatory. https://gco.iarc.fr/today/data/factsheets/populations/784-united-arab-emirates-fact-sheets.pdf.

[4] Tseng M, Ho F, Leong YH, Wong LC, Tham IW, Cheo T, Lee AW (2020). Emerging radiotherapy technologies and trends in nasopharyngeal cancer. Cancer Commun (Lond).

[5] Ng WT, Chow JC, Beitler JJ (2022). Current radiotherapy considerations for nasopharyngeal carcinoma. Cancers (Basel).

[6] Lee AW, Ma BB, Ng WT, Chan AT (2015). Management of nasopharyngeal carcinoma: current practice and future perspective. J Clin Oncol.

[7] Loong HH, Ma BB, Chan AT (2008). Update on the management and therapeutic monitoring of advanced nasopharyngeal cancer. Hematol Oncol Clin North Am.

[8] Lee AW, Ng WT, Chan YH, Sze H, Chan C, Lam TH (2012). The battle against nasopharyngeal cancer. Radiother Oncol.

[9] Sun XS, Li XY, Chen QY, Tang LQ, Mai HQ (2019). Future of radiotherapy in nasopharyngeal carcinoma. Br J Radiol.

[10] Gong G, Guo Y, Yin Y (2016). Su-F-P-26: study of radiation dose evaluation for organs at risk using MRI in intensity modulated radiation therapy for nasopharyngeal carcinoma. Med Phys.

[11] Yao CY, Zhou GR, Wang LJ (2018). A retrospective dosimetry study of intensity-modulated radiotherapy for nasopharyngeal carcinoma: radiation-induced brainstem injury and dose-volume analysis. Radiat Oncol.

[12] Powell C, Schick U, Morden JP (2014). Fatigue during chemoradiotherapy for nasopharyngeal cancer and its relationship to radiation dose distribution in the brain. Radiother Oncol.

[13] Chua ML, Chua KL, Wee JT (2019). Coming of age of bevacizumab in the management of radiation-induced cerebral necrosis. Ann Transl Med.

[14] George P, Abdullah S, Rachman A (2021). Analysis of hypothyroidism development in post-radiotherapy nasopharyngeal cancer patients using survival trees. J Phys Conf Ser.

[15] Zhao Z, Lan Y, Bai S (2013). Late-onset radiation-induced optic neuropathy after radiotherapy for nasopharyngeal carcinoma. J Clin Neurosci.

[16] Parsons JT, Bova FJ, Mendenhall WM, Million RR, Fitzgerald CR (1996). Response of the normal eye to high dose radiotherapy. Oncology (Williston Park).

[17] Low WK, Burgess R, Fong KW, Wang DY (2005). Effect of radiotherapy on retro-cochlear auditory pathways. Laryngoscope.

[18] Chan SH, Ng WT, Kam KL (2009). Sensorineural hearing loss after treatment of nasopharyngeal carcinoma: a longitudinal analysis. Int J Radiat Oncol Biol Phys.

